# The Role of Insulin-like Growth Factor 2 (IGF-2) in Periodontal Regeneration: A Systematic Review

**DOI:** 10.3390/medicina62010114

**Published:** 2026-01-05

**Authors:** Karina Natalie Kuntjoro, Yuniarti Soeroso, Fatimah Maria Tadjoedin, Nik Madihah Nik Azis, Nadhia Anindhita Harsas

**Affiliations:** 1 Periodontology Specialist Program, Faculty of Dentistry, Universitas Indonesia, Jakarta 10430, Indonesia; karina.kuntjoro@gmail.com; 2 Department of Periodontology, Faculty of Dentistry, Universitas Indonesia, Jakarta 10430, Indonesia; yuniarti_22@yahoo.co.id (Y.S.); fatimah.tadjoedin@ui.ac.id (F.M.T.); 3 Department of Restorative Dentistry, Universiti Kebangsaan Malaysia, Kuala Lumpur 50300, Malaysia; nikmadihah@ukm.edu.my

**Keywords:** insulin-like growth factor-2, guided bone regeneration, periodontal regeneration

## Abstract

*Background and Objectives*: Periodontal disease, characterized by progressive destruction of tooth-supporting tissues, often results in substantial alveolar bone loss, necessitating regenerative interventions such as guided bone regeneration (GBR). Insulin-like growth factor 2 (IGF-2) has emerged as a promising biomolecule for periodontal regeneration because of its osteogenic and immunomodulatory properties. *Materials and Methods*: A comprehensive literature search was conducted across five electronic databases (Scopus, ScienceDirect, PubMed, Wiley, and EBSCO). Studies examining the use of IGF-2 in periodontal or alveolar bone regeneration, including randomized controlled trials, animal studies, and in vitro experiments, were included. *Results*: Three studies met the inclusion criteria. In vitro, IGF-2 was associated with enhanced osteogenic differentiation and mineralization of mesenchymal stem cells, along with upregulation of key osteogenic markers. In animal models, IGF-2 treatment was associated with increased osteogenesis, greater bone volume, and a shift in macrophage polarization toward a less inflammatory phenotype. However, heterogeneity in study designs, protocols, and outcome measures limited direct comparisons. *Conclusions*: In vitro, IGF-2 was associated with enhanced osteogenic differentiation and mineralization of mesenchymal stem cells, accompanied by upregulation of key osteogenic markers. In animal models, IGF-2 treatment was associated with increased osteogenesis, greater bone volume, and a shift in macrophage polarization toward a less inflammatory phenotype.

## 1. Introduction

Periodontal disease is a complex, chronic inflammatory condition that involves the tooth-supporting tissues, including the periodontal ligament, cementum, and alveolar bone [1,2]. The World Health Organization (WHO) reports that 60% of adults worldwide suffer from periodontitis, with 24% of them experiencing severe periodontal conditions [3]. In Indonesia, the prevalence of severe periodontal disease among individuals aged over 15 years is 19.6%, ranking fourth highest in Southeast Asia [4]. 

The primary etiological factor of periodontitis is microbial biofilm, which initiates gingival inflammation, bleeding on probing (BoP), attachment loss, and progressive destruction of the alveolar bone. In advanced cases, extensive alveolar bone loss may result in tooth loss, thereby compromising mastication, oral function, and aesthetics [5]. Alveolar bone, like other bones in the body, undergoes remodeling through osteoclastic resorption and osteoblastic formation. Tooth loss due to periodontitis is often accompanied by irreversible alveolar bone resorption [6]. Zhao et al. reported that 0.56–1.38 mm of buccal and lingual bone loss can occur within six months following tooth extraction [7].

Dental implants are a standard tooth replacement option, but the risk of peri-implantitis and associated bone loss often necessitates bone augmentation to ensure implant stability [8]. One of the most used techniques for this purpose is Guided Bone Regeneration (GBR), which aims to increase bone volume in edentulous and peri-implant areas [9]. Based on the concept of Guided Tissue Regeneration (GTR), introduced by Nyman et al., GBR has evolved with the development of various graft materials and membranes to support alveolar bone regeneration, incorporating tissue engineering approaches that combine scaffolds, cells, and growth factors [10]. The gold standard for bone grafting is autogenous bone harvested from the patient; however, its limited availability has led to the development of alternatives, such as allografts, xenografts, and alloplasts. Despite their availability, non-autogenous materials typically lack osteoinductive potential [9]. Previous studies have explored adding growth factors to scaffolds to enhance bone regeneration [11,12,13]. Common growth factors used in periodontal tissue regeneration include fibroblast growth factor (FGF), platelet-derived growth factor (PDGF), vascular endothelial growth factor (VEGF), bone morphogenic protein (BMP), and Insulin-like growth factor (IGF).

For soft tissue regeneration, FGF, PDGF, and VEGF play essential roles, while BMP and IGF are crucial for bone formation. BMP-2 is the most widely used osteoinductive molecule in clinical practice. However, it requires high doses and may cause side effects, including ectopic bone formation and potential cancer risk, raising concerns about long-term safety and cost [12]. Another key growth factor in bone regeneration is the IGF family (IGF-1 and IGF-2), which regulates cell proliferation, differentiation, matrix production, and skeletal development. Although IGF-1 and IGF-2 are structurally similar, each plays a distinct role in bone metabolism. IGF-1 is vital after birth for supporting bone growth and tissue repair throughout life. In contrast, IGF-2 is mainly active during embryonic and fetal development, with its expression decreasing significantly after birth. Despite its lower levels in adults, IGF-2 remains essential for regulating stem cell activity, promoting osteogenic differentiation, and modulating inflammation [13,14].

**Figure 1:**
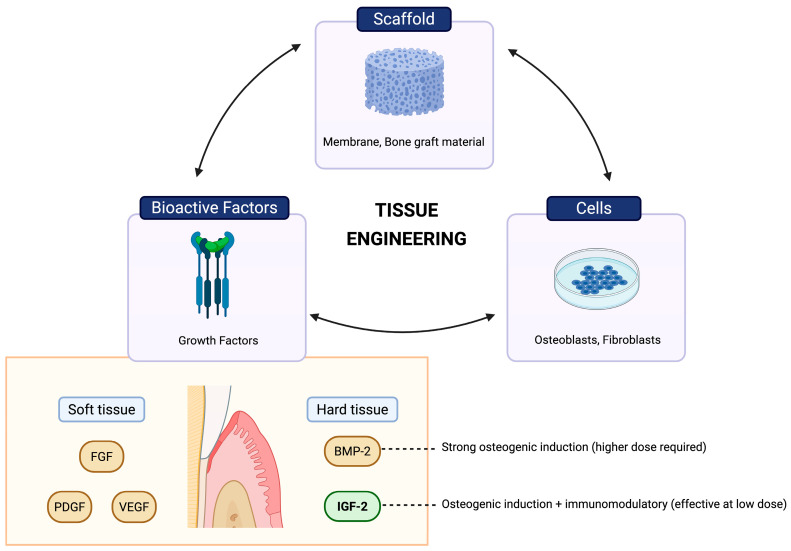
Schematic illustration of the interaction among scaffolds, cells, and bioactive factors during periodontal regeneration. Soft tissue regeneration is supported by FGF, PDGF, and VEGF, whereas hard tissue regeneration involves BMP-2 and the IGF family (IGF-1 and IGF-2). BMP-2 shows predominant osteoinductive activity requiring higher doses, while IGFs are associated with osteogenic and immunomodulatory effects reported at lower doses in preclinical models (created with BioRender.com).

Recent studies show that IGF-2 can enhance osteoblast differentiation and bone regeneration while reducing inflammation at low, non-toxic concentrations (5–20 ng/mL) [13,14]. This dual regenerative and anti-inflammatory activity makes IGF-2 a promising candidate for alveolar bone healing and periodontal tissue repair [15]. An overview of periodontal regeneration and the roles of these growth factors is presented in Figure 1. Given the growing interest in tissue-engineering strategies for periodontal therapy, this systematic review aims to evaluate the current evidence on the effects of IGF-2, alone or in combination with grafts or membrane materials, and to examine its safety, regenerative potential, and limitations for future clinical application in periodontal regeneration.

## 2. Materials and Methods

This systematic review was conducted in accordance with the Preferred Reporting Items for Systematic Reviews and Meta-Analyses (PRISMA) guidelines [16]. A comprehensive literature search was performed in Scopus, PubMed, ScienceDirect, Wiley Online Library, and EBSCO, covering publications from January 2015 to May 2025. The search strategy used a combination of Medical Subject Headings (MeSH) and free-text terms related to Insulin-like Growth Factor 2 and periodontal or alveolar bone regeneration, linked with Boolean operators (“AND”, “OR”). The full search strings were:

- Scopus, ScienceDirect, Wiley Online Library, and EBSCO: (“Insulin-like Growth Factor 2” OR “IGF-2”) AND (“periodontal regeneration” OR “alveolar bone regeneration”).
- PubMed: (“Insulin-Like Growth Factor II” [MeSH] OR “Insulin-like Growth Factor 2” OR “IGF-2”) AND (“periodontal regeneration” OR “alveolar bone regeneration”).

Only peer-reviewed studies published in English and available in full text were included. Gray literature, conference abstracts, preprints, and non-peer-reviewed sources were excluded. The search was last updated on 5 May 2025. The review protocol was prospectively registered in the Open Science Framework (OSF) under ID: osf.io/7vr6e.

The research question was formulated using the PICO framework (Population, Intervention, Comparison, Outcome) to explore the effects of Insulin-like Growth Factor 2 (IGF-2) on periodontal and alveolar bone regeneration compared with other growth factors or no treatment. The detailed PICO criteria used to guide study eligibility are presented in Table 1.

The study selection process was conducted manually using Microsoft Excel version 16.101.3 (Microsoft Corporation, Redmond, WA, USA)**.** After removing duplicates, four reviewers independently screened the titles and abstracts of all identified studies, followed by full-text evaluation of potentially eligible articles. Any disagreements were resolved through discussion to reach consensus, and a fifth reviewer was consulted when consensus could not be achieved. Additional details related to the population or model (including species, defect characteristics, and sample size), the intervention (such as formulation, dosage, application method, and duration), and outcome measures and main findings will also be recorded. 

Given the limited number and substantial heterogeneity of eligible preclinical studies, a quantitative meta-analysis was not performed. Instead, this systematic review was conducted as a qualitative synthesis in accordance with PRISMA guidelines.

The risk of bias in animal and in vitro studies was assessed by three reviewers using SYRCLE (Systematic Review Centre for Laboratory Animal Experimentation) and QUIN (Quality In vitro Studies) risk-of-bias tools, respectively [17,18]. The risk-of-bias evaluation, along with all other stages of the review process, including literature screening, full-text assessment, and data extraction, was conducted independently but was not blinded, as the reviewers were aware of the study authors, affiliations, and publication sources.

## 3. Results

### 3.1. Research Identification and Selection

**Figure 2:**
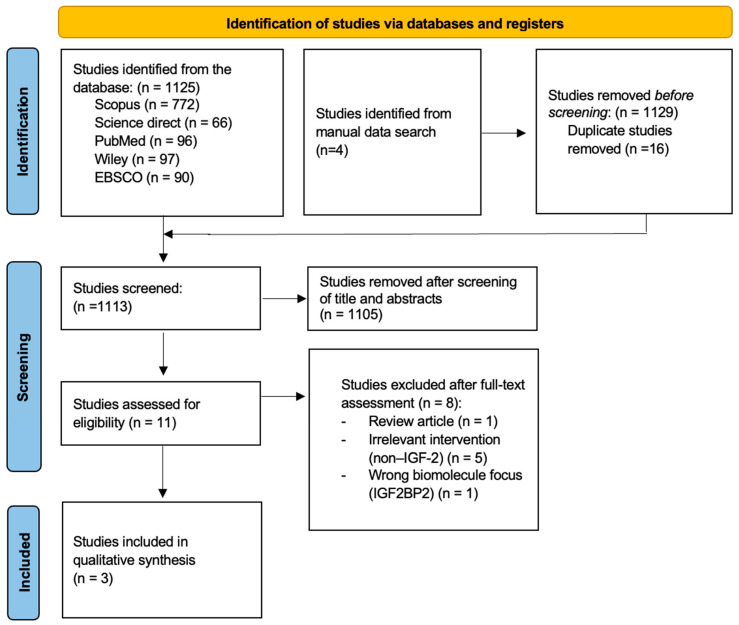
PRISMA flow diagram.

The study selection process followed the PRISMA guidelines, as illustrated in the flowchart in Figure 2. Research identification began with a comprehensive search across five electronic databases: Scopus, ScienceDirect, PubMed, Wiley, EBSCO, and other relevant sources. The search utilized a combination of keywords such as “IGF-2,” “periodontal regeneration,” and “alveolar bone regeneration.” A total of three studies were included in the final qualitative synthesis.

The literature search across five electronic databases yielded 1125 records, comprising 772 from Scopus, 66 from ScienceDirect, 96 from PubMed, 97 from Wiley, and 90 from EBSCO. An additional four records were identified through a manual search, bringing the total to 1129. After removing 16 duplicate records, 1113 records remained for title and abstract screening. Of these, 1105 records were excluded as they did not meet the inclusion criteria. Eleven full-text articles were assessed for eligibility, and eight were excluded due to an incompatible study design. Consequently, three studies were included in the final qualitative synthesis. These included the studies by Lee et al. [19], Wang et al. [20], and Diao et al. [21], which investigated the regenerative effects of IGF-2 and related molecules on periodontal and alveolar bone using in vitro and in vivo models, as summarized in Table 2 and Table 3.

### 3.2. Risk of Bias Assessment

**Figure 3:**
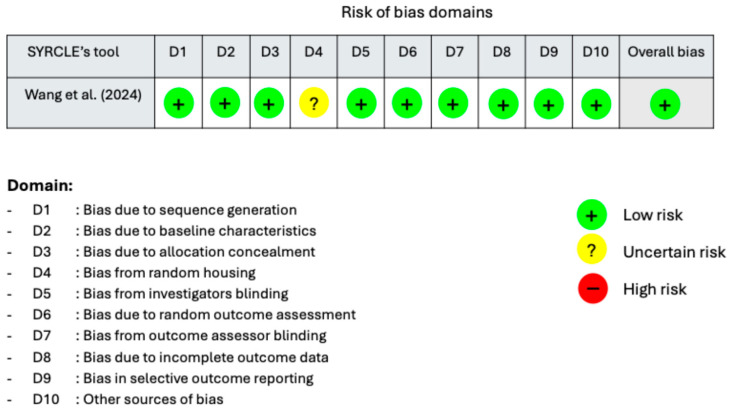
The Results of the Risk of Bias Assessment for in vivo studies using SYRCLE’s RoB tool [20].

The in vivo studies were evaluated using SYRCLE’s Risk of Bias (RoB) tool, which is adapted from the Cochrane RoB tool and modified to address biases specific to animal intervention studies. This tool comprises ten domains, with judgments categorized as low, unclear, or high risk of bias [17]. The in vivo studies by Wang et al. [20] were generally assessed as having a low risk of bias, supported by clearly defined experimental designs and predefined outcomes. However, the study had limitations regarding random housing, which was either unclear or not reported (Figure 3).

**Figure 4:**
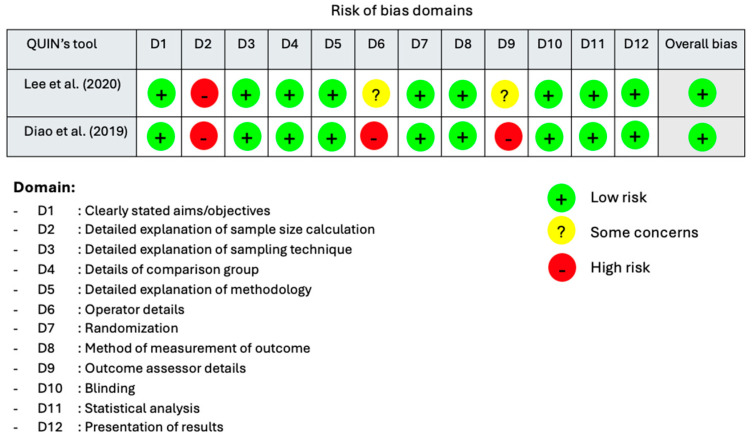
The Results of the Risk of Bias Assessment for in vitro studies using QUIN’s RoB tool [19,21].

In contrast, the in vitro studies were evaluated using QUIN’s RoB tool, which includes 12 domains. Assessment results were classified as low risk of bias, some concerns, or high risk of bias [18]. The in vitro studies by Lee et al. [19] and Diao et al. [21] were rated as having low risk of bias, although both had limitations in sample size calculation. In Lee et al.’s study, author contributions and assessor details were provided but not described in detail. Conversely, in Diao et al.’s study, these details were not reported at all (Figure 4).

### 3.3. Characteristics of Included Studies

The included studies varied in terms of experimental design, species, cell types, interventions, and outcomes measured. Specifically, one study [20] employed animal models of periodontitis, whereas the others used in vitro cell cultures [19,21]. Different cell sources, such as human bone marrow-derived mesenchymal stem cells (hBM-MSCs), stem cells from apical papilla (SCAPs), and periodontal disease models in mice, were evaluated. The primary outcomes assessed included osteogenic differentiation, alveolar bone regeneration, inflammation markers, and osteoclastogenesis [19,20,21].

### 3.4. In Vivo Studies

Wang et al. [20] reported that local IGF-2 administration in a ligature-induced mouse model of periodontitis was associated with anti-inflammatory and regenerative effects. High-dose IGF-2 (10 ng/mL) reduced levels of pro-inflammatory cytokines IL-1β, IL-6, and TNF-α by approximately 50% (*p* < 0.01) and decreased iNOS expression, suggesting attenuation of M1 macrophage polarization. Micro-CT and histological analyses further showed a dose-dependent increase in alveolar bone volume (BV/TV) in IGF-2–treated animals (*p* < 0.05), indicating enhanced bone regeneration under experimental conditions. At the molecular level, IGF-2 treatment increased expression of osteogenic markers, including RUNX2 and OPN (40–50%; *p* < 0.05), suggesting stimulation of osteogenic activity at the defect site.

Mechanistic findings further indicate that these effects may be associated with modulation of the cGAS/STING–NF-κB signaling pathway in experimental models. The cGAS/STING pathway plays a key role in innate immune responses and has been implicated in inflammation-associated bone loss in periodontal disease. Sustained activation of this pathway is linked to pro-inflammatory macrophage polarization and impaired tissue repair. Additionally, conditioned-medium experiments indicate that IGF-2 exposure may indirectly promote osteogenic differentiation of periodontal ligament fibroblasts under inflammatory conditions, supporting a potential dual role in modulating inflammation and promoting tissue regeneration.

### 3.5. In Vitro Studies

Lee et al. [19] investigated the effects of IGF-2 on hBM-MSCs cultured on a DBBM scaffold. IGF-2 supplementation at 10–100 ng/mL significantly improved cell viability compared to untreated controls (*p* < 0.05), while preserving normal fibroblast-like morphology. Although ALP activity showed only a mild and statistically insignificant increase at Days 7 and 14 (*p* > 0.05), mineralization was significantly enhanced at these time points (*p* < 0.05). Additionally, BGLAP expression was upregulated at both concentrations, indicating improved late-stage osteogenic maturation on the scaffold surface.

In contrast, Diao et al. [21] showed that low-dose IGF-2 (5 ng/mL) significantly promoted osteo-/dentinogenic differentiation in SCAPs, as indicated by higher ALP activity, increased calcium deposits, and enhanced expression of genes involved in mineralized tissue formation. Additionally, IGF-2 upregulated neurogenic markers and stimulated cell proliferation, while proteomic analysis revealed increased secretion of proteins related to osteogenesis, neurogenesis, and cell growth. This indicates a broader regulatory role as a niche-derived factor supporting SCAP function.

Collectively, these findings indicate that IGF-2 may promote regeneration across different mesenchymal stem cell types. Its effects may vary depending on the cell source and microenvironment, with SCAPs showing stronger osteogenic and neurogenic responses, while hBM-MSCs benefit most when combined with a scaffold.

### 3.6. Suitability for Meta-Analysis

Due to substantial methodological heterogeneity across studies, including differences in cell sources, experimental approaches, measured outcomes, and interventions, a meta-analysis was not feasible. Instead, a narrative synthesis was conducted to integrate and interpret the results qualitatively.

### 3.7. Concluding Summary of Findings

Collectively, the available evidence suggests that IGF-2 may be associated with periodontal tissue regeneration and the modulation of inflammatory processes under experimental conditions. However, the current body of evidence is limited by the small number of eligible studies and substantial methodological heterogeneity. Consequently, further well-designed, standardized preclinical studies, followed by early-phase clinical investigations, are required before definitive conclusions can be drawn or quantitative synthesis can be undertaken.

## 4. Discussion

Periodontal regeneration involves the coordinated renewal of alveolar bone, periodontal ligament, and cementum, driven by precisely controlled cellular, molecular, and inflammatory mechanisms. This systematic review highlights the emerging therapeutic potential of IGF-2 as a bioactive molecule that can synergistically influence these mechanisms. Across the included studies, IGF-2 was associated with hard-tissue regenerative outcomes and immune response modulation, suggesting that it may warrant further investigation in the context of periodontal regeneration.

In vitro studies indicate that IGF-2 is associated with increased osteogenic activity in dental-derived stem cells. Specifically, IGF-2 treatment was linked to enhanced mineralized nodule formation and upregulation of osteogenic markers such as BGLAP, RUNX2, and OPN [19,21]. These observations suggest a potential role for IGF-2 across multiple stages of osteogenic differentiation. In addition, increased expression of neurogenic-associated markers, including βIII-tubulin and nestin, was reported in SCAPs after IGF-2 exposure [21,22]. While these findings point to broader cellular effects, the functional significance of neurogenic marker expression in periodontal regeneration remains uncertain and warrants further investigation.

**Figure 5:**
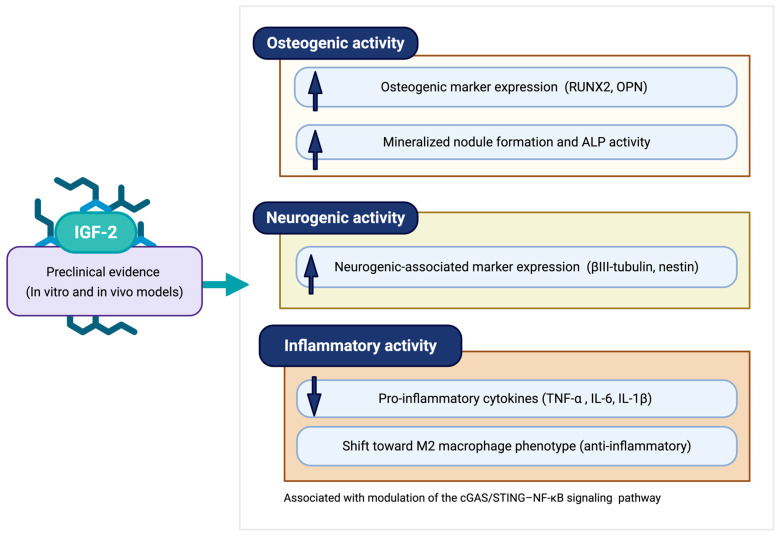
Schematic summary of preclinical evidence describing IGF-2–associated biological activities relevant to periodontal regeneration. In vitro and in vivo studies indicate increased osteogenic marker expression, mineralized nodule formation, and neurogenic-associated marker expression, alongside modulation of inflammatory activity characterized by reduced pro-inflammatory cytokine expression and a shift toward anti-inflammatory (M2) macrophage polarization. These effects are observed in experimental models and are associated with modulation of the cGAS/STING–NF-κB signaling pathway [19,20,21] (created with BioRender.com).

Evidence from a single in vivo study suggests that IGF-2 administration may be associated with reduced inflammatory activity and enhanced alveolar bone regeneration under experimental periodontitis conditions. Wang et al. reported decreased levels of pro-inflammatory cytokines (TNF-α, IL-6, and IL-1β), suppression of M1 macrophage polarization, and increased bone volume fraction following local IGF-2 treatment [20]. These effects were accompanied by upregulation of osteogenic markers at the defect site. Mechanistic analyses implicated modulation of the cGAS/STING–NF-κB signaling pathway, suggesting that IGF-2 may influence innate immune signaling rather than exert a nonspecific anti-inflammatory effect [20,23]. However, as these findings are derived from a single animal model, their reproducibility and translational relevance remain to be confirmed. Overall, available in vitro and in vivo evidence suggests that IGF-2 may be associated with osteogenic and immunomodulatory effects in experimental models of periodontal disease, although conclusions are limited by the small number of heterogeneous preclinical studies (Figure 5).

Compared with established growth factors such as PDGF and FGF-2, which are primarily associated with angiogenesis and cellular proliferation, IGF-2 may exert combined osteogenic and immunomodulatory effects in preclinical settings [24]. Nevertheless, direct comparative evidence is lacking, and conclusions regarding relative efficacy should be interpreted cautiously. Present data do not justify applying these findings to clinical settings.

Several important limitations must be acknowledged. Only three eligible preclinical studies were identified, and substantial heterogeneity was observed in experimental models, cell types, dosing regimens, delivery strategies, and outcome measures. This heterogeneity precluded meta-analysis and limited the ability to draw definitive conclusions. Furthermore, no clinical studies evaluating IGF-2 in periodontal regeneration have been published to date.

Risk-of-bias assessment using the SYRCLE and QUIN tools revealed consistent methodological shortcomings across the included studies. Inadequate reporting of randomization procedures, unclear allocation concealment, lack of blinding, and insufficient sample size justification raise concerns about potential selection, performance, and detection bias. These limitations may have influenced effect estimates and necessitate cautious interpretation of the reported regenerative outcomes.

Future research should prioritize well-designed, adequately powered in vivo studies with standardized methodologies and outcome measures. Early-phase clinical trials will be necessary to evaluate safety, dosing, delivery approaches, and long-term outcomes before IGF-2 can be considered for clinical application. In summary, while preclinical evidence suggests that IGF-2 may be associated with bone regenerative and immunomodulatory processes, the current evidence base remains limited and insufficient to support clinical translation at this stage.

## 5. Conclusions

Based on available preclinical evidence, IGF-2 is associated with enhanced osteogenic activity, modulation of inflammatory responses, and increased expression of neurogenic markers in experimental models of periodontal and alveolar bone regeneration. However, the current evidence is limited to a small number of heterogeneous in vitro and in vivo studies, with variability in experimental design, cell types, dosing strategies, and outcome measures. Consequently, the findings should be interpreted with caution. Further well-designed, standardized in vivo studies, followed by early-phase clinical investigations, are required to clarify the safety profile, optimal dosage, delivery strategies, and potential clinical relevance of IGF-2 in periodontal regenerative therapy.

## Figures and Tables

**Table 1:** PICO Description.

Population (P)	Intervention (I)	Comparison (C)	Outcome (O)
Human/animal-derived cells cultured under periodontal disease-like conditions or on biomaterials/scaffolds.	Application of IGF-2 alone or combined with a biomaterial/scaffold for periodontal or alveolar bone regeneration.	Control groups without IGF-2 or treated with other growth factors.	Primary outcome: Bone and soft tissue regeneration (clinical, histological, and radiographic outcomes). Secondary outcome: Cellular response (osteogenesis, angiogenesis, extracellular matrix formation) parameters.

**Table 2:** Summary of Included Studies.

Study	Year	Study Design	Sample Size	Cell Type	Intervention	Main Outcomes
Lee et al. [19]	2020	In vitro	N/A	hBM-MSCs cultured on deproteinized bovine bone mineral (DBBM)	IGF-2 (10, 100 ng/mL)	Effects of IGF-2 on osteogenic activity and mineralization.
Wang et al. [20]	2024	In vivo	9 mice divided into 3 groups (*n* = 3/group)	Murine ligature-induced periodontitis	Local IGF-2 administration (control, low dose 5 ng/mL, high dos e 10 ng/mL)	Anti-inflammatory and osteogenic effects, focusing on macrophage polarization and bone regeneration.
Diao et al. [21]	2019	In vitro	10 human-impacted third molar teeth	SCAPs	IGF-2 treatment (0, 5 ng/mL)	Osteo/dentinogenic and neurogenic differentiation

**Table 3:** Interstudy Comparison.

Study	Intervention	Markers	Main Outcomes	Findings
Lee et al. [19]	IGF-2 + DBBM scaffold	Cell viability: Live/Dead staining (qualitative) + CCK-8 assay (quantitative) Alkaline phosphatase (ALP): early osteogenic marker Bone gamma-carboxyglutamate protein (BGLAP)/osteocalcin gene: late osteogenic marker Alizarin Red staining: mineralization marker	Enhanced osteogenic differentiation, increased mineralization	Cell viability: ↑ significantly at 10–100 ng/mL (*p* < 0.05). ALP activity: Mild ↑, but not significant at Days 7 and 14 (*p* > 0.05). Mineralization: Significant ↑ at Days 7–14 (*p* < 0.05). BGLAP expression: ↑ at 10–100 ng/mL, indicating advanced osteogenic maturation.
Wang et al. [20]	IGF-2 administration	Pro-inflammatory cytokines: TNF-α, IL-6, IL-1β Macrophage polarization markers: M1 (iNOS), M2 (flow cytometry) Osteogenic markers: RUNX2, OPN (RT-qPCR & Western blot) Bone regeneration: BV/TV (micro-CT)	Reduced inflammation, improved alveolar bone regeneration	High-dose IGF-2 (10 ng/mL): ↓ IL-1β, IL-6, TNF-α by ~50% (*p* < 0.01) ↓ M1 macrophages (iNOS), M2 macrophages remained stable ↑ RUNX2 & OPN by 40–50% (*p* < 0.05) ↑ BV/TV significantly in a dose-dependent manner (*p* < 0.05)
Diao et al. [21]	IGF-2 treatment	ALP activity: early osteogenic marker Alizarin Red staining & calcium assay: mineralization marker) Osteo/dentinogenic genes: DSPP, DMP1, BSP, OCN, OPN (RT-qPCR) Neurogenic markers: Nestin, βIII-tubulin	Increased osteo-/dentinogenic differentiation	ALP activity: ↑ significantly at 5 ng/mL (*p* < 0.05). Mineralization: ↑ calcium deposition and Alizarin Red staining (*p* < 0.01). Osteo/dentinogenic genes: DSPP, DMP1, BSP, OCN, OPN significantly ↑. Neurogenic markers: Nestin, βIII-tubulin ↑ and ↑ proliferation indicating neural regenerative potential.

Note: ↑ indicates increase; ↓ indicates decrease.

## Data Availability

Additional data related to this article are available from the corresponding author upon reasonable request.

## Abbreviations

The following abbreviations are used in this manuscript:

ALP	Alkaline phosphatase
BGLAP	Bone gamma-carboxyglutamate protein (osteocalcin gene)
BMP	Bone morphogenetic protein
BoP	Bleeding on probing
BV/TV	Bone Volume over Total Volume
cGAS	cyclic GMP–AMP synthase
DBBM	Deproteinized bovine bone mineral
DSPP	Dentin sialo phosphoprotein
FGF	Fibroblast growth factor
GBR	Guided bone regeneration
GTR	Guided tissue regeneration
hBM-MSCs	Human bone marrow–derived mesenchymal stem cells
IGF	Insulin-like growth factor
IL	Interleukin
iNOS	Inducible nitric oxide synthase (M1 macrophage marker)
M1	Macrophage pro-inflammatory phenotype
M2	Macrophage anti-inflammatory phenotype
MESH	Medical Subject Headings
OCN	Osteocalcin
OPN	Osteopontin
OSF	Open Science Framework
PDGF	Platelet-derived growth factor;
PICO	Population, intervention, comparison, and outcome
PRISMA	Preferred Reporting Items for Systematic Reviews and Meta-Analyses
QUIN	Quality assessment tool for in vitro studies
RCT	Randomized controlled trial
RoB	Risk of bias
RUNX2	Runt-related transcription factor 2
SCAPs	Stem cells from apical papilla
STING	Stimulator of interferon genes
SYRCLE	Systematic Review Centre for Laboratory Animal Experimentation
TNF-α	Tumor necrosis factor-alpha
VEGF	Vascular endothelial growth factor
WHO	World Health Organization
